# Dietary Supplementation of a Multi-Strain Probiotic Increases Muscle Mass in Pigs

**DOI:** 10.3390/ijms27104381

**Published:** 2026-05-14

**Authors:** Shu-Hua Hsu, Ting-Yu Lee, Chao-Wei Huang, Bishnu Prasad Bhattarai, Yu-I Pan, Yi-Chu Liao, Hsiao-Tung Chang, Hsin-Hsuan Huang, Jin-Seng Lin, Xin Zhao, Jai-Wei Lee

**Affiliations:** 1 Department of Tropical Agriculture and International Cooperation, National Pingtung University of Science and Technology, Pingtung 912301, Taiwan; p11122001@mail.npust.edu.tw (S.-H.H.); cwhuang@mail.npust.edu.tw (C.-W.H.); bbhattarai987@uga.edu (B.P.B.); 2 SYNBIO TECH Inc., Kaohsiung 821011, Taiwan; tingyu.lee@synbiotech.com.tw (T.-Y.L.); yc.liao@synbiotech.com.tw (Y.-C.L.); hsiaotung@synbiotech.com.tw (H.-T.C.); thera_huang@synbiotech.com.tw (H.-H.H.); jslin@synbiotech.com.tw (J.-S.L.); 3 Department of Poultry Science, University of Georgia, Athens, GA 30602, USA; 4 Department of Veterinary Medicine, College of Veterinary Medicine, National Pingtung University of Science and Technology, Pingtung 912301, Taiwan; yuipan@mail.npust.edu.tw; 5 Department of Animal Science, McGill University, Québec, QC H9X 3V9, Canada; xin.zhao@mcgill.ca

**Keywords:** muti-strain probiotics, muscle mass, pig production

## Abstract

Pork production is closely linked to skeletal muscle growth and anabolic processes. This study investigated the effects of dietary supplementation with a multi-strain probiotic (*Lactiplantibacillus plantarum*, *Streptococcus thermophilus*, and *Bacillus subtilis*) on the growth performance, carcass traits, gut microbiota, and potential signaling pathways in growing pigs. A total of 144 weaning piglets (28 days old) were randomly allocated to two groups and fed diets with or without probiotics (0.1%) for 18 weeks. Pigs fed with probiotics showed significantly improved feed efficiency (*p* < 0.05) and greater muscle mass in the loin eye, arm shoulder, and blade shoulder regions. Microbiome analysis revealed significant enrichment of short-chain fatty acid (SCFA)-producing taxa, including *Acidaminococcus*, *Allisonella*, *Dialister*, and *Megasphaera*, alongside an increased cecal butyrate level in pigs fed probiotics. Integrated fecal microbiome and serum metabolomics analysis demonstrated that the metabolite profile was substantially altered by the supplementation of probiotics. Additionally, serum insulin levels, expression of the bile acid receptor *tgr5*, and upstream genes in the PI3K/Akt/mTOR pathway (*igf1r*, *insr*, and *pi3k*) were significantly upregulated (*p* < 0.05). Collectively, these results suggest that a multi-strain probiotic supplementation may be a promising strategy for improving muscle deposition and feed efficiency in commercial pig production.

## 1. Introduction

Improving growth performance and reducing production costs have long been research interests for the swine industry. Since skeletal muscle accounts for approximately 40–50% of total body mass in pigs [1], optimizing muscle development is essential for improving carcass traits and overall production efficiency. Skeletal muscle hypertrophy is fundamentally driven by protein synthesis and protein degradation, a balance regulated by a complex intracellular signaling network, such as the myostatin signaling pathway [2], Wnt/β-catenin signaling [3] and PI3K/Akt/mTOR pathway. Among the known regulators, the PI3K/Akt/mTOR pathway serves as a central hub for skeletal muscle homeostasis. In this pathway, PI3K and Akt are activated by IGF-1 and insulin, which leads to the activation of mTOR (specifically mTORC1) [4]. Concurrently, activated Akt is able to inhibit protein degradation by phosphorylating forkhead box O (FOXO) transcription factors [5]. Taken together, the PI3K/Akt/mTOR signaling pathway plays a pivotal role in determining the net muscle mass due to its dual-functional property in promoting protein synthesis and suppressing protein degradation simultaneously [6].

A healthy gut microbiota is beneficial for host metabolism, immunity, and nutrient absorption. Microbial-derived metabolites, including short-chain fatty acids (SCFAs) and secondary bile acids, can translocate into the systemic circulation and serve as distal signals to regulate skeletal muscle growth [7]. For instance, the production of SCFAs, particularly butyrate, improves insulin sensitivity and enhances Akt phosphorylation (Thr308) in skeletal muscle [8], and has been demonstrated to promote muscle mass and function through the activation of PI3K/Akt/mTOR signaling in rodent models of sarcopenia [9,10]. In addition, certain microbial strains transform primary bile acids (BAs) into secondary BAs, which serve as molecular bridges to regulate metabolic function such as muscle protein turnover [7,11]. The composition of the gut microbiota also directly influences the bile acid pool available for systemic signaling [12], and metabolically active microbial taxa at the cecal mucosa are associated with feed efficiency in pigs [13]. Therefore, dietary supplementation of probiotics to enhance gut health could be a feasible strategy for improving skeletal muscle development [14,15]. The inclusion of *Lactobacillus plantarum* TWK10 and *Bacillus subtilis* in the diet was able to increase muscle mass in mice [16] and breast muscle in broilers [17], respectively. Moreover, *Lactobacillus paracasei* has been reported to decelerate age-related muscle loss by maintaining protein uptake and preserving mitochondrial function in a mouse model [18]. Given the distinct anatomical and physiological differences between rodents and pigs [19], however, the metabolic impact of probiotic supplementation may differ across these models.

The application of probiotics to swine production has been studied primarily in the contexts of growth performance, gut health, and disease prevention. A meta-analysis of 67 randomized controlled trials reported that probiotic supplementation improved the average daily gain and feed conversion ratio across growing pigs, with effects that varied by strain identity, production stage and breeds [20]. Beyond growth performance, probiotic supplementation has also been reported to positively affect carcass and meat quality traits in pigs, and *Lactobacillus reuteri* has been shown to upregulate the expression of amino acid transporters without examining its effects on muscle growth [21].

While most studies focused on single-strain probiotics, using multi-strain formulations has become a trend in the industry due to their broader synergistic benefits [20,22]. Nevertheless, the effects of using multi-strain probiotics are not always consistent, which could be attributed to the composition of probiotic strains and host responses [23]. Their specific effects on skeletal muscle development remain poorly documented in pigs. To address these gaps, the effects of supplementing SYNLAC LeanAd, a commercial multi-strain probiotic product (SYNBIO TECH INC., Kaohsiung, Taiwan), in diets on the growth performance, carcass quality, gut microbiota composition, SCFA profiles, and metabolic pathways related to muscle development were investigated in pigs from weaning to finishing.

## 2. Results

### 2.1. Growth Performance

As shown in Table 1, no significant differences in BW and BWG were observed between the two dietary treatments throughout the trial. However, inclusion of LeanAd in the diet significantly reduced AFI (*p* < 0.05) from 5 to 15 weeks, resulting in a significantly lower FCR (1.96 vs. 2.18) than that of the control. Moreover, the overall FCR for the LeanAd group over the period of 5 to 22 weeks was also significantly (*p* < 0.05) lower (2.33) in comparison to that of the control group (2.49).

### 2.2. Carcass Quality

Pigs fed with diets containing LeanAd had significantly (*p* < 0.05) increased loin eye area (65.18 vs. 53.20 cm^2^), arm shoulder (10.74 vs. 9.74 kg), and blade shoulder (5.66 vs. 4.97 kg) compared to those fed with the control diet (Table 2). However, no significant differences were found in live weight or slaughter percentage at 22 weeks of age between the two treatment groups. Additionally, meat quality traits including lean meat percentage, fat percentage, bone percentage, and pH of BF and LT at 1 and 24 h postmortem, as well as meat color, cooking loss, and drip loss, showed no significant differences between the two groups.

### 2.3. Intestinal Microbiota

To evaluate the impact of LeanAd on the pig gut microbiota, intestinal digesta samples were analyzed using 16S rRNA sequencing. At the phylum level, the control group exhibited a significantly higher relative abundance of *Campilobacterota* (*p* < 0.05) compared to the LeanAd group, while *Actinobacteriota* showed a modest increase in the LeanAd group (*p* < 0.05) (Table 3). At the genus level, *Acidaminococcus*, *Allisonella*, *Dialister*, *Erysipelotrichaceae*_UCG-006, *Lachnospiraceae*_ND3007 and *Megasphaera* were significantly abundant (*p* < 0.05) in the LeanAd group. In contrast, *Campylobacter*, *Lachnoclostridium* and *Moryella* were significantly more abundant in the control group (*p* < 0.05) (Table 4).

### 2.4. Serum Metabolomic Analysis

**Figure 1:**
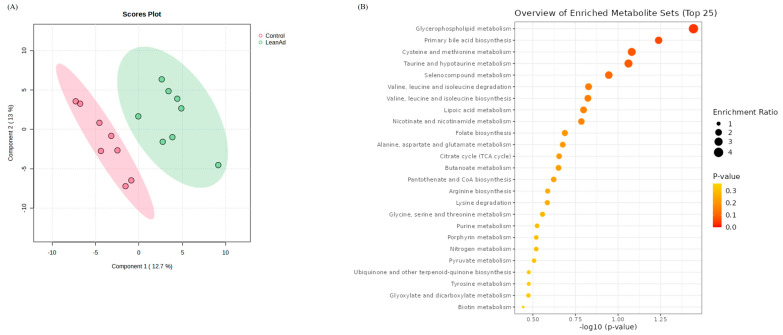
Multivariate statistical analysis of serum metabolites in control and LeanAd group. (**A**) PLS-DA plots are presented illustrating the distinct separation and key metabolic features underlying group-specific differences. (**B**) Metabolic pathway enrichment analysis of differentially expressed metabolites between the control and LeanAd groups. The bubble size represents the amount of the compound in the metabolic pathway according to the relative-betweenness centrality algorithm (*n* = 6 per treatment).

To assess the impact of LeanAd on metabolic alterations in pigs, a comprehensive analysis of plasma metabolites was conducted. A total of 1399 distinct peaks were identified, with 841 observed in positive ionization mode and 558 in negative ionization mode, as indicated by the metabolomic data. Results from PLS-DA showed a clear differentiation between the control and LeanAd groups, resulting in a model with high reliability and predictability (Figure 1A). Furthermore, functional annotation and metabolic pathway enrichment analysis using the Kyoto Encyclopedia of Genes and Genomes (KEGG) was performed for the differentially expressed metabolites. The analysis identified the top three significant pathways, which were associated with glycerophospholipid metabolism, primary bile acid biosynthesis, and the metabolism of cysteine and methionine (Figure 1B).

### 2.5. Integrative Analysis of Microbiome and Metabolome

**Figure 2:**
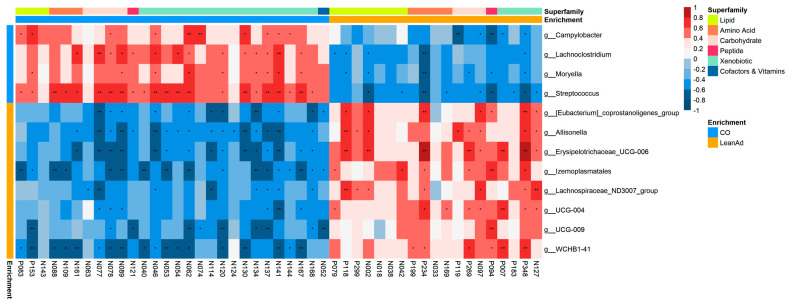
Correlation analysis between gut microbiota and serum metabolome. Spearman’s correlation heatmap showing associations between 12 bacterial genera and 47 discriminatory metabolites. The red squares indicate positive correlations, whereas the blue squares indicate negative correlations. The asterisk (*) indicates significant difference between groups (*n* = 6 per treatment, * *p* < 0.05, ** *p* < 0.01).

To investigate the interactions between the gut microbiota and serum metabolites, Spearman correlation analysis was performed across 12 bacterial genera and 47 significantly altered metabolites (VIP > 1.0; FDR-adjusted *p*-value < 0.05). The resulting correlation heatmap illustrated significant associations among microbial taxa and metabolites (Figure 2), which are listed in Supplementary Table S1. Metabolites were classified into functional subfamilies based on KEGG annotation, including lipids, amino acids, carbohydrates, peptides, cofactors/vitamins, and xenobiotics. The change in composition in the gut microbiome was associated with different systemic metabolites. In categories related to known nutrients, 34.29% (107 of the 312 pairs) of bacterial genera were significantly correlated (*p* < 0.05) with their corresponding metabolites. Among these, 55 pairs (51.40%) were positively correlated and 52 pairs (48.60%) were negatively correlated. These correlations indicate that LeanAD supplementation is associated with eight bacterial genera, which were linked to a distinct metabolite pool enriched in lipids and amino acids. In contrast, the control group was associated with fewer bacterial taxa and was characterized predominantly by xenobiotic metabolites.

Distinct genus-level associations were observed between groups. In the control group, *Campylobacter*, *Moryella* and *Lachnoclostridium* were dominant taxa, showing correlations with altered metabolites. *Campylobacter* and *Moryella* were significantly associated with lipid and carbohydrate metabolites, whereas *Lachnoclostridium* was linked to amino acids, carbohydrates, and peptides. In contrast, the LeanAD group exhibited unique associations: *Allisonella* was positively correlated with carbohydrate and lipid metabolites, while *Lachnospiraceae*_ND3007_group and *Erysipelotrichaceae*_UCG-006 were linked to lipids, amino acids, and carbohydrates. The details are listed in Supplementary Tables S2 and S3.

### 2.6. Short-Chain Fatty Acid (SCFA) Analysis

The concentrations of SCFAs in the cecum were quantified to evaluate the potential metabolic activity of the gut microbiota (Table 5). LeanAd supplementation was associated with a significant increase in cecal butyrate levels (20.95 vs. 27.46 μmol/g; *p* < 0.05). In contrast, no significant differences were observed in the concentrations of acetate or propionate between the LeanAd and the control groups.

### 2.7. Blood Hormone Analysis

The blood insulin concentration observed in the LeanAd group of pigs (11.35 mIU/mL) was significantly elevated compared to that in the control group (8.70 mIU/mL) (*p* < 0.01) (Table 6). Conversely, the levels of blood IGF-1 between the two groups were not statistically different.

### 2.8. Effect of mTOR-Related Gene Expression and Bile Acid Receptor in Muscle

**Figure 3:**
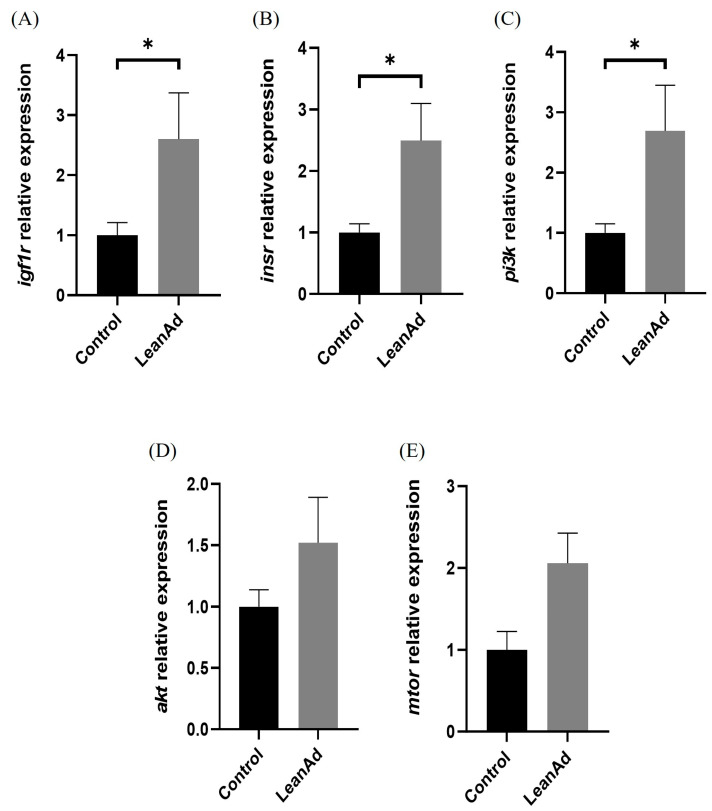
The relative expression levels of positive regulators of mTOR-related genes in swine muscle. (**A**) *igf1r*, (**B**) *insr*, (**C**) *pi3k*, (**D**) *akt*, and (**E**) *mtor*. Data are presented as mean ± SEM and were analyzed by Mann–Whitney U test. The asterisk (*) indicates significant difference between groups (*n* = 6 per treatment, *p* < 0.05).

**Figure 4:**
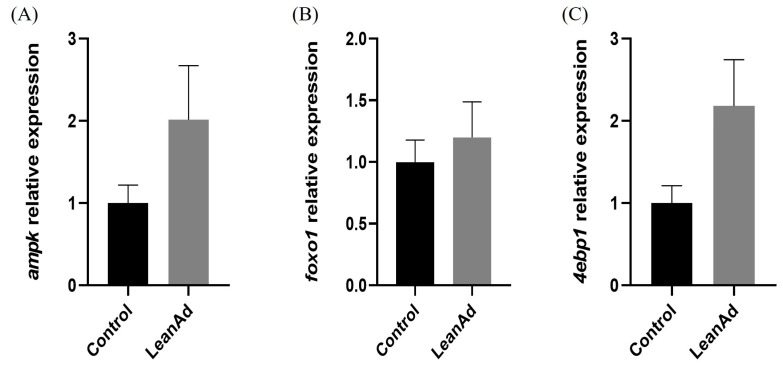
The relative expression levels of negative regulators of mTOR-related genes in swine muscle. (**A**) *ampk*, (**B**) *foxo1*, and (**C**) *4ebp1*. Data was analyzed by Mann–Whitney U test and are presented as mean ± SEM (n = 6 per treatment).

**Figure 5:**
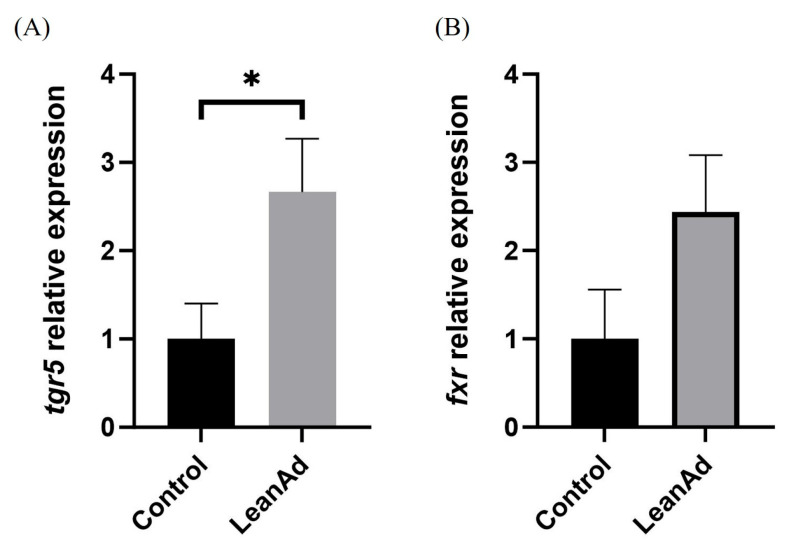
The relative expression levels of bile acid receptor genes in swine muscle. (**A**) tgr5, (**B**) fxr. Data are presented as mean ± SEM and were analyzed by Mann–Whitney U test. The asterisk (*) indicates significant difference between groups (*n* = 6 per treatment, *p* < 0.05).

The comparative analysis of mTOR-related gene expression in muscle tissues revealed that pigs from the LeanAd group indicated a statistically significant (*p* < 0.05) enhancement in the expression levels of positive regulators, specifically *igf1r*, *insr*, and *pi3k*, in contrast to the control group (Figure 3A–C). Conversely, regarding the expression of negative regulators associated with mTOR pathways (*4ebp1*, *foxo1*, and *ampk*), no significant differences were observed between the two treatment groups (Figure 4). In metabolite analysis, bile acid-related metabolites MDCA and GUDCA were present at relatively higher levels in pigs from the LeanAd group. Therefore, the expression of Farnesoid X Receptor (*fxr*) and *tgr5* in muscle were examined. In comparison with the control group, *tgr5* was significantly (*p* < 0.05) increased in the LeanAd group (Figure 5A). Nevertheless, the expressions of *fxr* were not different between the two treatment groups (Figure 5B).

## 3. Discussion

In terms of growth performance, supplementation with LeanAd significantly improved FCR, especially in the grower phase, which was associated with reduced AFI during weeks 5–15. However, differences in BWG between the two groups were not statistically significant. It can be postulated that probiotic supplementation may enhance nutrient and energy utilization, so that feed intake is decreased. These findings are consistent with our previous study using the same multi-strain probiotic formulation containing *L. plantarum*, *S. thermophilus*, and *B. subtilis*, in which supplementation of probiotics improved FCR by enhancing gut integrity and shifting the gut microbiome during the weaning stage of piglets (4–8 weeks old) [24].

Although pigs from the LeanAd group had similar growth rates when compared to those in the control group, muscle masses in the loin eye area (LEA; the cross-section of the LD muscle), arm, and blade shoulder were increased. LEA is a reliable predictor of carcass composition and quality traits [25]. LEA was measured at the 10th–11th rib interface according to the *Taiwan Pork Carcass Cutting Manual* of the National Animal Industry Foundation (NAIF) standard [26]. Within the consistent measurement framework of this study, the significantly greater LEA in pigs from the LeanAd group reflected an increase in longissimus dorsi muscle cross-sectional area. Our results suggest that dietary supplementation of LeanAd has the potential to enhance muscle deposition. These observations align with those from studies using rodents and broilers, indicating that probiotic supplementation positively influences skeletal muscle mass [16,17]. To the best of our knowledge, this is the first study demonstrating that dietary supplementation of probiotics is able to increase muscle mass in pigs.

Metabolic factors such as insulin regulation play a critical role in muscle development. Insulin promotes glucose uptake in skeletal muscle and maintains its integrity [27]. Moreover, higher insulin levels are linked to increased LEA and muscle fiber cross-sectional area in pigs [28]. In pigs from the LeanAd group, elevated serum insulin levels were observed along with increased expression of insulin-like growth factor 1 receptor (*igf1r*) and insulin receptor (*insr*). Frampton et al. (2020) indicated that SCFAs may enhance muscle protein synthesis by improving insulin sensitivity and regulating glycogenesis [29]. In line with these findings, our results also demonstrated a correlation between insulin regulation and “gut metabolism” in pigs fed with LeanAd. Specifically, the elevated serum insulin concentrations in these pigs were associated with a significant increase in cecal butyate levels. Cecal butyrate, predominately produced from the microbial fermentation of dietary fibers, is a key energy source that maintains gut barrier integrity and metabolic health [30]. The cecal butyrate content was 31% higher in pigs from the LeanAd group compared to those from the control group. SCFAs in the large intestine can provide 6.9–12.0% of maintenance energy in 48 kg pigs and 4.8–12.0% in 89 kg pigs [31]. In addition, microbiota-derived butyrate prevents age-related skeletal muscle loss through the PI3K/Akt/mTOR pathway in the rodent model [9]. Collectively, these findings suggest that increased butyrate may enhance protein synthesis and muscle mass in LeanAd-fed pigs.

The PI3K/Akt/mTOR pathway integrates signals from nutrients, growth factors, and microbial metabolites to regulate protein synthesis and cellular growth [10]. Our analysis found that the expression of upstream *pi3k*, but not *akt* and *mtor*, was significantly increased in pigs from the LeanAd group. It has been demonstrated that the activation of this signaling pathway is primarily driven by post-translational modifications rather than changes in total mRNA abundance [32]. In the mouse model, insulin signaling was enhanced by increasing the phosphorylation of insulin receptor substrate-1 and Akt in the skeletal muscle [8]. Moreover, expressions of negative regulators, including *ampk*, *foxo1*, and *4ebp1*, remained unchanged, supporting the notion that enhanced protein synthesis can drive muscle hypertrophy even when basal protein degradation is unaffected [32].

The metabolic efficiency observed in the pigs from the LeanAd group may be associated with both host-derived metabolites and gut microbiome modulation. A stable microbial community has been known to stimulate efficient nutrient utilization, which can contribute to increased host growth potential and lean meat transformation [21]. Our results indicated that LeanAd supplementation significantly altered the gut microbiota composition in 22-week-old pigs. Dominant taxa included *Allisonella*, *Erysipelotrichaceae*_UCG-006, and *Lachnospiraceae*_ND3007, all of which are positively correlated with lipid metabolites, including GUDCA, MDCA, and icosabutate. The secondary bile acids GUDCA and MDCA were enriched in pigs from the LeanAd group, along with significant upregulation of the bile acid receptor *tgr5* in muscle tissue. These findings are consistent with the established role of *tgr5* activation in enhancing glucose utilization and promoting muscle hypertrophy [33].

At the phylum level, Firmicutes was the most abundant phylum in both groups. However, pigs fed with LeanAd exhibited a significantly lower richness of *Campilobacterota*, which was attributable to the reduced abundance of the genus *Campylobacter*. Given that *Campylobacter* abundance is negatively associated with feed efficiency [13], a reduction in this abundance is associated with improved feed efficiency, consistent with reduced metabolic competition for energy substrates. In contrast, LeanAd pigs showed enrichment of SCFA-producing genera within the *Veillonellaceae* family, including *Dialister*, *Allisonella*, and *Megasphaera*. This suggests a more robust conversion of organic substrates into beneficial SCFAs [34,35]. Therefore, shifting the gut microbiome may positively correlate with improved feed efficiency and may be associated with cecal butyrate levels.

The integrative role of serum metabolites in facilitating microbiota–host interactions revealed distinct profiles between two groups, with the LeanAd group showing significant enrichment in glycerophospholipid metabolism, primary bile acid biosynthesis, and cysteine and methionine metabolism (the top three). Glycerophospholipids are the major components of cell membranes. The upregulation of this pathway suggests active membrane remodeling that supports rapid tissue accretion. Moreover, the modulation of glycerophospholipid metabolism has been reported to influence hepatic lipid metabolism [36]. Bile acids, the end products of cholesterol metabolism, facilitate lipid utilization and also function as potent signaling molecules that regulate host metabolic pathways [7]. Alterations in cysteine and methionine metabolism have been positively correlated with carcass weight and muscle glycogen storage [37]. These upregulated pathways may play a role in lipid utilization and amino acid metabolism, which have beneficial effects on improved feed efficiency and muscle mass observed in pigs from the LeanAd group.

In conclusion, our results indicate that dietary supplementation with LeanAd is able to improve feed efficiency and skeletal muscle growth in commercial pig production. Molecular pathways connecting gut microbial modulation and skeletal muscle deposition were also proposed. Specifically, pigs fed with LeanAd showed upregulated expression of upstream genes in the PI3K/AKT/mTOR signaling pathway and alterations in bile acid metabolism consistent with anabolic activation. Although further research is needed to clarify the underlying mechanisms and establish causation, these findings suggest that the application of a multi-strain probiotic may be a feasible approach to improve production efficiency and carcass traits of commercial pigs.

## 4. Materials and Methods

### 4.1. Ethical Approval Statement

This animal study was approved by the Institutional Animal Care and Use Committee of the National Pingtung University of Science and Technology (NPUST), Pingtung, Taiwan (approval no. NPUST-112-102; approved on 27 September 2023).

### 4.2. Animals and Experimental Design

A total of 144 crossbred weaning piglets (Landrace × Yorkshire × Duroc), aged 28 days, were individually ear-tagged for tracking and identification. The piglets were randomly assigned to eight floor pens, with 18 piglets allocated to each pen. The animals were further grouped according to sex, with four pens designated for barrows and four for gilts, with an average weight of 7.92 ± 1.01 kg. These pens were divided into 2 treatment groups (4 pens each, 2 barrow pens and 2 gilt pens). The pigs were raised in a controlled environment within a closed housing system for a duration of 18 weeks. Each pen was equipped with two water nipples and a feeder, providing ad libitum access to water and feed throughout the experiment. Piglets were individually weighed using an electronic scale at 4, 15 and 22 weeks of age to determine body weight (BW) and body weight gain (BWG). Additionally, average feed intake (AFI) was recorded during each growth phase. The feed conversion ratio (FCR) was calculated as the total feed intake divided by the total weight gain per pen. At the end of the feeding trial (22 weeks of age), a total of six pigs (three males and three females) were randomly selected from each treatment (n = 6) to analyze blood hormones levels, fecal SCFAs, gut microbiome composition, and carcass quality.

### 4.3. Experiment Diets

Pigs in this trial were fed with diets according to their physiological stages, including prestarter (5–6 weeks of age), starter (7–12 weeks of age), grower (13–18 weeks of age), and finisher (19–22 weeks of age). The nutritional composition of the experimental diets, detailed in Table 7, met or exceeded the nutritional requirements for pigs as established by the National Research Council [38]. The dietary treatments for all stages consisted of a basal diet (Control) and the basal diet supplemented with 0.1% SYNLAC LeanAd (SYNBIO TECH INC., Kaohsiung, Taiwan), a commercially available multi-strain probiotic formulation comprising *Lactiplantibacillus plantarum* LP28 (1.0 × 10^8^ CFU/g), *Streptococcus thermophilus* ST30 (1.0 × 10^6^ CFU/g), and *Bacillus subtilis* STCC 0015 (3.0 × 10^8^ CFU/g).

### 4.4. Blood and Fecal Sampling

Blood samples were collected from the jugular vein and placed in BD Vacutainer^®^ SST™ Tubes (BD Co., Ltd., Franklin Lakes, NJ, USA) for blood biochemical analysis. Blood samples were centrifuged at 1000× *g* for 10 min at 4 °C for serum isolation. The isolated serum samples were then stored at −20°C until further analysis. Fresh fecal samples were collected as previously described with modifications [39]. Briefly, pigs were gently partitioned in their pens using sorting panels. When pigs spontaneously defecated, fresh fecal samples were collected by experienced personnel wearing sterile gloves and directly placed into sterile plastic bags. The initial portion of the feces was discarded, and approximately 3–5 g of the middle segment was collected into a fecal collection tube. Samples were immediately placed on dry ice for transport and subsequently stored at −80 °C until further analysis.

### 4.5. Carcass Quality

All carcass evaluation procedures followed the *Taiwan Pork Carcass Cutting Manual* of NAIF [26]. During the slaughtering process, the Carometec UltraFom 300 (UF-300) ultrasound scanner (SFK Technology A/S, Herlev, Denmark) was used on the slaughter line to scan the 3rd–4th lumbar vertebrae and the last rib at a position 7 cm from the dorsal midline. The instrument analyzed backfat thickness and estimated carcass lean meat percentage. Following the ultrasonic measurements, the pig carcasses were transported to the pre-cooling room, where detailed carcass data were collected. Once the carcasses achieved a core temperature of 5 °C, they were subsequently segmented, deboned, trimmed, and packaged.

Each carcass was split along the abdominal and dorsal midline. The left half was further sectioned into arm shoulder, blade shoulder, neck, *Longissimus dorsi* (loin, LL), belly, and *Biceps femoris* (ham, BF). Subcutaneous fat and bones were separated from each portion and weighed individually to determine their relative proportions in the carcass. The remaining tissue was then used to calculate the lean percentage. According to the NAIF manual and Taiwan’s commercial trading standard, hot carcass weight is defined as the weight after exsanguination and removal of viscera and reproductive organs, retaining the head, skin, feet, tail, and leaf lard. Slaughter percentage (dressing rate) was calculated as hot carcass weight divided by live weight.

Loin eye area (LEA) was determined on the excised LL muscle following the NAIF manual. The muscle was transversely sectioned at the interface of the 10th and 11th ribs, and the cross-sectional outline was traced onto tracing paper. The enclosed area was then quantified using a Portable Area Meter (LI-3000, LI-COR Biosciences, Lincoln, NE, USA) and expressed in cm^2^. In Taiwan’s commercial slaughter workflow, the *Longissimus dorsi* is removed from the carcass prior to chilling to accommodate downstream line processing. Additionally, LL muscle samples at the level of the 1st to 4th lumbar vertebrae were excised into four 2 cm thick slices and stored at 4 °C for 24 h in plastic bags to determine drip loss (DL) and cooking loss (CL). The DL was calculated as the difference between initial and final weights, expressed as a percentage. Furthermore, muscle samples were cooked to an internal core temperature of 75 °C for 40 min; the weight difference before and after cooking was recorded to ascertain CL, also expressed as a percentage [40]. In addition, *Longissimus thoracis* (LT) muscle samples (6 g) were collected from the left half of the carcass, quickly frozen in liquid nitrogen, and stored at −80 °C for subsequent qPCR quantification and Western blot analysis.

### 4.6. 16S rRNA Gene Sequencing Analysis

The fecal samples (1 g) were suspended in 9 mL saline solution (0.85% NaCl), from which 200 μL of the mixture was used for the extraction of total bacterial DNA using the DNA kit (Qiagen, Valencia, CA, USA). The concentration of final extracted DNA was evaluated using a Qubit fluorometer (ThermoFisher, Waltham, MA, USA). The amplification of the 16s rRNA gene was performed from V3 to V4 regions by PCR using bacterial universal primers of 319F (5′-CCTACGGGNGGCWGCAG-3′) and 806R (5′-GACTACHVGGGTATCTAATCC-3′), with each sample assigned a unique eight-base sequence barcode. The pooled amplicon libraries were then sequenced on the Illumina MiSeq platform (Illumina, San Diego, CA, USA). Subsequently, quality filtering of the sequences was conducted using the q2-demux plugin, followed by denoising with DADA2 (via q2-dada2) [41] within QIIME 2 version 2020.8 [42], resulting in the generation of amplicon sequence variants (ASVs). The ASVs were mapped to the SILVA database (release version 138) and classified with a 99% similarity threshold using the q2-feature-classifier plugin in QIIME 2 [43]. Subsequent analyses of microbiota composition were performed utilizing the phyloseq package (R version 1.40.0). Differences in community structure among dietary groups were assessed by permutational multivariate analysis of variance (PERMANOVA) using the vegan package (R version 2.6.4).

### 4.7. Analyses of Blood Hormones

The serum samples were analyzed for insulin and insulin-like growth factor 1 (IGF-1) using commercially available spectrophotometric kits (MBS2700907, MBS701022, MyBioSource, Inc., San Diego, CA, USA). The detection limits were 3.12 ng/mL for IGF-1 and 2 uIU/mL for insulin. The intra- and inter-assay coefficients of variation were 10% and 12% for IGF-1, respectively, and <15% for insulin, respectively. All protocols were performed according to the manufacturer’s instructions.

### 4.8. Serum Metabolomic Analysis

Serum metabolites were extracted by mixing 100 μL of serum with 800 μL of ice-cold methanol containing an internal standard. Following extraction, the supernatant was dried under a gentle stream of nitrogen and reconstituted in 200 μL of methanol (equivalent to twice the original serum volume). The reconstituted samples were filtered through a 0.22 μm membrane and stored at −20 °C until analysis. Chromatographic separation was performed on a Dionex Ultimate 3000 UHPLC system (Thermo Fisher Scientific, Waltham, MA, USA) equipped with quaternary pumps and coupled to a Q Exactive Plus hybrid quadrupole-Orbitrap mass spectrometer. Quality control (QC) samples were injected at regular intervals (every 8 samples). All raw data were processed using Compound Discoverer 3.3.0 (Thermo-Fisher Scientific, Waltham, MA, USA). For metabolite identification, the detected peaks were annotated based on the similarity to the authentic metabolite spectra in the LWHK metabolite database (Leeuwenhoek Laboratories Co., Ltd., Taipei, Taiwan) or reference spectra in online databases (mzCloud, metabolika, Chemspider). Metabolite similarity was determined based on precursor mass error (<5 ppm), isotope pattern matching, and, when applicable, fragmentation spectra. For quality control, pooled QC samples were injected at regular intervals (one QC injection every eight test samples), and metabolites were retained only if their coefficient of variation (CV) across QC samples was <30%. Statistical significance was assessed using a two-tailed Student’s *t*-test on internal standard-normalized peak areas. To control for multiple comparisons, *p*-values were adjusted using the Benjamini–Hochberg procedure to calculate the false discovery rate (FDR). Differential metabolites were defined based on the dual criteria of variable importance in projection (VIP) > 1.0 from the partial least-squares discriminant analysis (PLS-DA) model and an FDR-adjusted *p*-value < 0.05. PLS-DA was performed using MetaboAnalyst 6.0 [44].

### 4.9. Analyses of Fecal SCFAs

The fecal sample (1 g) was homogenized in 5 mL of water. A 10% perchloric acid solution (KATAYAMA CHEMICAL Co., Ltd., Osaka, Japan) was then added at a 9:1 ratio. The mixture was stored at 4 °C for 2 h and subsequently centrifuged at 10,000× rpm for 15 min. The supernatant was filtered using a 0.22 µm membrane filter (Millipore Japan Ltd., Tokyo, Japan). The analytical standards of all SCFAs were analyzed under the same HPLC conditions to establish standard calibration for quantitation of SCFAs in samples as described [24].

### 4.10. Real-Time Quantitative PCR

The total RNA was obtained from LT muscle samples using QIAGEN RNeasy^®^ Mini kit (cat no. 74106, QIAGEN N.V., Hilden, Germany) and stored at −80 °C. Total RNA (1 μg/μL) was reverse transcribed into cDNA using the High-Capacity cDNA Reverse Transcription kit with RNase inhibitor (Applied Biosystems, Waltham, MA, USA, cat. no. 4374966) following the manufacturer’s protocol (25 °C for 10 min, 37 °C for 120 min, 85 °C for 5 min, and a final hold at 4 °C). The gene expression levels of *igf1r*, *insr*, *pi3k*, *akt*, *mtor*, *4ebp1*, *foxo1*, *ampk*, *tgr5* and *fxr* were determined (n = 6 for each treatment group) via quantitative real-time PCR. The reactions were performed on a QuantStudio 3 real-time PCR system (Thermo Fisher Scientific, Waltham, MA, USA) with Maxima SYBR Green qRT-PCR Master Mix (2x) (Thermo Scientific, Waltham, MA, USA). The relative gene expression levels were calculated using the 2^−ΔΔCt^ method. β-actin was used as the housekeeping gene for normalization. The primer sequences are shown in Table 8 and the qPCR conditions followed a previously published protocol.

### 4.11. Statistical Analysis

Experimental data were statistically analyzed using a General Linear Model (GLM) in GraphPad Prism software version 9.4.1 (Boston, MA, USA). For growth performance (BW, BWG, AFI, and FCR), each pen served as the experimental unit (n = 4 per treatment), with individual pig weights averaged within each pen. For intestinal morphology, SCFAs, and carcass quality, the individual pig was considered the experimental unit (n = 6 per treatment), and one pen was included as a random effect to account for the shared environment. All the results are presented as mean ± standard error of the mean (SEM). Differences in the means between treatment groups were assessed using an independent samples *t*-test. Permutational multivariate analysis of variance (PERMANOVA) was analyzed using vegan: Community Ecology Package (R package version 2.5-7). The differences were considered statistically significant at *p* < 0.05. Spearman’s rank correlation coefficients were calculated to assess associations between microbiota and metabolites, with results visualized as a heatmap.

## 5. Conclusions

Improved feed efficiency and carcass traits were observed in pigs fed with LeanAd in the present study. Analysis of fecal microbiota and serum metabolites revealed that there is a molecular linkage between microbiota-driven metabolic shifts and skeletal muscle deposition. Moreover, modulation of the PI3K/AKT/mTOR signaling pathway and bile acid markers were also observed in pigs fed with LeanAd. While direct evidence remains to be established, these findings suggest that multi-strain probiotic supplementation may facilitate muscle development through microbiota-mediated metabolic regulation, offering new insights into the application of probiotics for improving production efficiency and certain carcass traits in commercial pig production.

## Figures and Tables

**Table 1:** Effects of LeanAd in production performance of pigs ^1^.

Items ^2^	Control	LeanAd	SEM	*p*-Value
BW, kg				
4 weeks	7.97	7.89	0.12	0.612
15 weeks	63.66	62.50	0.58	0.404
22 weeks	114.40	114.50	0.05	0.954
BWG, kg				
5–15 weeks	55.68	54.59	0.55	0.400
16–22 weeks	50.85	52.08	0.62	0.223
5–22 weeks	106.4	106.5	0.05	0.946
AFI, kg^2^				
5–15 weeks	119.21 ^a^	102.21 ^b^	7.10	<0.001
16–22 weeks	144.79	142.36	0.15	0.516
5–22 weeks	264.00	244.57	8.60	0.2638
FCR				
5–15 weeks	2.18 ^a^	1.96 ^b^	0.11	<0.001
16–22 weeks	2.87	2.77	0.04	0.255
5–22 weeks	2.49 ^a^	2.33 ^b^	0.08	0.009

^1^ *n* = 4 per treatment. ^2^ BW, Body weight; BWG, body weight gain; AFI, average feed intake; and FCR, feed conversion ratio. SEM: Standard error of means for each item. ^a,b^ Mean values in the same row with a different superscript differ significantly (*p* < 0.05).

**Table 2:** Effects of LeanAd on physical characteristics of meat in pigs at week 22 ^1^.

	Control	LeanAd	SEM	*p*-Value
Live weight, kg	115.67	115.95	2.76	0.920
Carcass weight, kg	98.78	99.28	1.93	0.803
Slaughter, %	85.45	86.64	0.95	1.000
Lean meat, %	50.12	53.10	1.40	0.132
Fat, %	13.83	10.85	1.60	0.132
Bone, %	15.10	15.35	0.47	0.589
Fat-to-lean ratio (F:L)	0.28	0.21	0.04	0.081
Loin eye area, cm^2^	53.20 ^b^	65.18 ^a^	5.24	0.044
Backfat thickness, cm^2^	1.98	1.65	0.27	0.243
Arm shoulder, kg	9.74 ^b^	10.74 ^a^	0.41	0.036
Blade shoulder, kg	4.97 ^b^	5.66 ^a^	0.16	0.001
Neck, kg	2.30	2.40	0.30	0.746
Loin, kg	7.64	8.02	0.29	0.240
Belly, kg	9.33	9.52	0.57	0.751
Ham, kg	15.38	16.24	0.68	0.234
Cooking loss, %	31.68	32.32	1.07	0.591
Dripping loss, %	4.93	6.05	0.76	0.175
pH_1hr_				
BF1	6.26	6.27	0.14	0.953
BF2	6.20	6.22	0.12	0.904
LT1	6.20	6.27	0.09	0.462
LT2	6.22	6.31	0.09	0.338
pH_24hr_				
BF1	5.84	5.77	0.06	0.289
BF2	5.85	5.76	0.07	0.228
LT1	5.86	5.76	0.08	0.223
LT2	5.85	5.72	0.08	0.112

^1^ *n* = 6 per treatment. SEM: Standard error of means for each item. ^a,b^ Mean values in the same row with a different superscript differ significantly (*p* < 0.05).

**Table 3:** Abundance of microbial taxa at the phylum level between the two groups at 22 weeks old ^1^.

	Control ^2^	LeanAd ^2^	SEM	*p*-Value
*Actinobacteriota*	0.94	1.75	0.43	0.068
*Bacteroidota*	12.09	15.36	2.76	0.198
*Campilobacterota*	0.11 ^a^	0.01 ^b^	0.04	0.001
*Cyanobacteria*	0.11	0.13	0.02	0.347
*Desulfobacterota*	0.24	0.09	0.09	0.410
*Euryarchaeota*	0.55	0.45	0.13	0.443
*Firmicutes*	80.56	76.16	3.48	0.198
*Proteobacteria*	0.18	0.14	0.04	0.291
*Spirochaetota*	3.74	4.01	1.11	0.977

^1^ *n* = 6 per treatment. ^2^ Data are mean values in the same row. SEM: Standard error of means for each item. ^a,b^ Mean values in the same row with a different superscript differ significantly (*p* ˂ 0.05).

**Table 4:** Abundance of microbial taxa at the genus level between the two groups at 22 weeks old ^1^.

	Control ^2^	LeanAd ^2^	SEM	*p*-Value
*[Eubacterium]_ruminantium_group*	0.10	0.03	0.034	0.089
*Acidaminococcus*	0.00 ^b^	0.01 ^a^	0.003	0.028
*Allisonella*	0.00 ^b^	0.02 ^a^	0.005	0.024
*Asteroleplasma*	0.01	0.01	0.004	0.045
*Butyricicoccus*	0.08	0.14	0.078	0.089
*Campylobacter*	0.05 ^a^	0.01 ^b^	0.012	0.007
*Colidextribacter*	0.10	0.04	0.026	0.052
*Collinsella*	0.07	0.15	0.043	0.060
*Dialister*	0.11 ^b^	0.63 ^a^	0.258	0.039
*Erysipelotrichaceae_UCG-006*	0.00 ^b^	0.04 ^a^	0.012	0.010
*Lachnoclostridium*	0.07 ^a^	0.02 ^b^	0.015	0.008
*Lachnospiraceae_ND3007_group*	0.02 ^b^	0.04 ^a^	0.013	0.045
*Libanicoccus*	0.00	0.01	0.005	0.068
*Megasphaera*	0.30 ^b^	0.83 ^a^	0.197	0.008
*Moryella*	0.07 ^a^	0.05 ^b^	0.008	0.017
*UCG-009*	0.10	0.13	0.035	0.078

^1^ *n* = 6 per treatment. ^2^ Data are mean values in the same row. SEM: Standard error of means for each item. ^a,b^ Mean values in the same row with a different superscript differ significantly (*p* ˂ 0.05).

**Table 5:** Effect of LeanAd on the contents of SCFAs in cecum of pigs at 22 weeks old ^1^.

	Control	LeanAd	SEM	*p*-Value
Acetate (μmol/g)	79.34	82.37	4.77	0.532
Propionate (μmol/g)	37.06	40.73	3.72	0.335
Butyrate (μmol/g)	20.95 ^b^	27.46 ^a^	2.92	0.038

^1^ *n* = 6 per treatment. SEM: Standard error of means for each item. ^a,b^ Mean values in the same row with a different superscript differ significantly (*p* ˂ 0.05).

**Table 6:** The effect of LeanAd on the IGF-1 and insulin in serum of pigs at 22 weeks old ^1^.

	Control	LeanAd	SEM	*p*-Value
IGF1, ng/mL	65.83	80.80	18.67	0.436
Insulin, mIU/mL	8.70 ^b^	11.35 ^a^	0.80	0.006

^1^ *n* = 6 per treatment. SEM: Standard error of means for each item. ^a,b^ Mean values in the same row with a different superscript differ significantly (*p* ˂ 0.05).

**Table 7:** Ingredients and chemical composition of basal diets (as fed basis, %).

Items	Week 4–6	Week 7–12	Week 13–18	Week 19–22
Extruded corn	56.20	10.00	-	-
Corn	-	50.43	70.90	68.53
Full-fat soybean meal	12.50	10.00	-	-
Soybean meal, dehulled	-	20.00	25.00	24.00
Fermented soybean meal	10.00	-	-	
Wheat bran	-	-	-	4.50
Whey powder	7.50	2.50	-	-
Fat powder	5.00	-	-	-
Soybean oil	1.00	4.00	1.50	0.40
Plasma meal	4.00	-	-	-
Monocalcium phosphate	0.80	0.80	0.80	0.80
Limestone	0.80	0.80	0.80	0.80
Acidifier	0.30	0.20	0.05	-
Phytase	0.03	0.03	0.03	0.03
Salt	0.40	0.40	0.40	0.40
Choline chloride, 50%	0.15	0.10	0.10	0.10
Lysine, 78.8%	0.40	0.40	0.20	0.18
Methionine, 99%	0.20	0.10	0.06	0.04
Threonine, 99%	0.10	0.08	0.08	0.07
Tryptophan, 98.5%	0.08	0.01	-	-
Vitamins and minerals	0.54	0.15	0.15	0.15
Total	100.00	100.00	100.00	100.00
**Calculated Composition**				
Metabolic energy, kcal/kg	3621.22	3566.11	3409.41	3296.72
Crude protein, %	18.81	18.23	17.22	17.23
Crude fat, %	8.10	8.30	4.40	3.40
Crude fiber, %	2.51	3.15	3.13	3.43
Lysine, %	1.40	1.28	1.04	1.02
Methionine + Cystine, %	0.84	0.70	0.73	0.64
Threonine, %	0.91	0.77	0.65	0.71
Tryptophan, %	0.30	0.22	0.20	0.20
Calcium, %	0.61	0.58	0.56	0.56
Total phosphorus, %	0.61	0.53	0.51	0.54
Available Phosphorus, %	0.37	0.32	0.24	0.25

**Table 8:** Primer sequences of target and reference genes.

	Gene	Primer Sequence	References
1	*igf1r*	F: 5′-ATG GAT CAC AAA GCC CTC GG-3′	[45]
		R: 5′-CTG CCG CCA CTA CTA CTA CG-3′	
2	*akt*	F: 5′-TCC AGC TTG AGG TCC CGA TA-3′	[45]
		R:5′-GCT CTT CTT CCA CCT GTC CC-3′	
3	*mtor*	F: 5′-GGG GTT TGG ATC AGG GTC TG-3′	[45]
		R: 5′-GAC TCA TCC GCC CCT ACA TG-3′	
4	*insr*	F: 5′-CAC TGG CTA TCG CAT TGA GC-3′	[46]
		R: 5′- CCT GCC ACA TCA AGT GAA CG-3′	
5	*pi3k*	F: 5′-GAC TGT GGG ATT GAG ACG-3′	[46]
		R: 5′- ACC CGA GTA AGA ATG TGC-3′	
6	*ampk*	F: 5′-CGA CGT GGA GCT GTA CTG CTT-3′	[47]
		R: 5′-CAT AGG TCA GGC AGA ACT TGC-3′	
7	*4ebp1*	F: 5′-CCG GAA GTT CCT AAT GGA GTG T-3′	[47]
		R: 5′-GGT TCT GGC TGG CAT CTG T-3′	
8	*foxo1*	F: 5′-CGGCATCATCTTCATCGTC-3′	[12]
		R: 5′-CTGTCCTCCCACTCCAGGTA-3′	
9	*tgr5*	F: 5′-TGCTGTCCCTCATCTCATTGG-3′	[48]
		R: 5′-TGTGTAGCGATGATCACCCAG-3′	
10	*fxr*	F: 5′-TATGAACTCAGGCGAATGCCTGCT-3′	[48]
		R: 5′- ATCCAGATGCTCTGTCTCCGCAAA-3′	
11	*β-actin*	F: 5′-CCA GCA CGA TGA AGA TCA AGA-3′	[49]
		R: 5′-AAT GCA ACT ACA GTC CGC CTA-3′	

## Data Availability

Raw sequence files supporting the findings of this article are deposited at the NCBI Sequence Read Archive (SRA) database with project accession number PRJNA1437894.

## Supplementary Materials

The following supporting information can be downloaded at https://www.mdpi.com/article/10.3390/ijms27104381/s1.

## Abbreviations

The following abbreviations are used in this manuscript:

FOXO	phosphorylating forkhead box O
SCFAs	short-chain fatty acids
BSH	bile salt hydrolase
BAs	bile acids
MDCA	murideoxycholic acid
GUDCA	glycoursodeoxycholic
TGR5	Takeda G-protein-coupled receptor 5
BW	body weight
BWG	body weight gain
AFI	average feed intake
FCR	feed conversion ratio
LL	*Longissimus dorsi*
DL	drip loss
CL	cooking loss
LT	*Longissimus thoracis*
IGF-1	insulin-like growth factor 1
QC	quality control
CV	coefficient of variation
FDR	false discovery rate
VIP	variable importance in projection
PLS-DA	partial least-squares discriminant analysis
GLM	general linear model
SEM	standard error of the mean
PERMANOVA	permutational multivariate analysis of variance
BF	biceps femoris
KEGG	Kyoto encyclopedia of genes and genomes
FXR	Farnesoid X Receptor
NAIF	The National Animal Industry Foundation, Taiwan
