# Supplementary material for: Dietary Supplementation of a Multi-Strain Probiotic Increases Muscle Mass in Pigs
Source: Int J Mol Sci. 2026 May 14;27(10):4381. doi: 10.3390/ijms27104381 (PMC13207453; doi:10.3390/ijms27104381)
Supplement: Supplementary file 1 [file ijms-27-04381-s001.zip › ijms-4301388-supplementary.pdf]

Supplementary Information

Dietary Supplementation of a Multi-Strain Probiotic Increases Muscle Mass in Pigs

Shu-Hua Hsu [1,†], Ting-Yu Lee [2,†], Chao-Wei Huang [1], Bishnu Prasad Bhattarai [1,3], Yu-I Pan [4], Yi-Chu Liao [2], Hsiao-Tung Chang [2], Hsin-Hsuan Huang [2], Jin-Seng Lin [2], Xin Zhao [5] and Jai-Wei Lee [1,*]

[1] Department of Tropical Agriculture and International Cooperation, National Pingtung University of Science and Technology, Pingtung 912301, Taiwan
[2] SYNBIO TECH Inc., Kaohsiung 821011, Taiwan
[3] Department of Poultry Science, University of Georgia, Athens, GA 30602, USA
[4] Department of Veterinary Medicine, College of Veterinary Medicine, National Pingtung University of Science and Technology, Pingtung 912301, Taiwan
[5] Department of Animal Science, McGill University, Québec, QC H9X 3V9, Canada
* Correspondence: joeylee@mail.npust.edu.tw; Tel.: +886-8-770-3202 (ext. 6417)
† These authors contributed equally to this work.

Supplementary Table S1 List of metabolites code and full name

Compound code	Name
P083	1-Tetradecylamine
P153	Lauryldimethylamine oxide
N143	12-HSA
N088	4-Hydroxyphenylpyruvate
N109	4-Hydroxyphenylpyruvate
N161	(4E)-4-[(2Z)-2-{[1-Carboxy-3-(methylsulfinyl)propyl]imino}ethylidene]-1,2,3,4-tetrahydro-2,6-pyridinedicarboxylic acid
N063	4-Hydroxy-5-(2-hydroxy-2-propanyl)-2-methylbicyclo[3.1.0]hex-2-yl hexopyranoside
N077	4-Hydroxy-5-(2-hydroxy-2-propanyl)-2-methylbicyclo[3.1.0]hex-2-yl hexopyranoside
N078	2-(3,4-Dihydroxyphenyl)-5,7-dihydroxy-4-oxo-4H-chromen-3-yl 4-O-β-D-glucopyranosyl-β-D-glucopyranosiduronic acid
N089	2-(3,4-Dihydroxyphenyl)-5,7-dihydroxy-4-oxo-4H-chromen-3-yl 4-O-β-D-glucopyranosyl-β-D-glucopyranosiduronic acid
N121	[3-(3,4-methylenedioxyphenyl)-2-(mercaptomethyl)-1-oxoprolyl]glycine
N040	7-benzyloxy-4-trifluoromethylcoumarin
N046	N-(6-Butyl-1,3-benzothiazol-2-yl)-2-thiophenecarboxamide
N053	penflutizide
N054	7DMA
N062	methyl N-(2,6-dichlorobenzyl)-3-[2-(2,6-dichlorophenyl)-6-quinolyl]-N-methylalaninate
N074	Versiconal
N114	N-(2-{[6-(2,6-Dichloro-3,5-dimethoxyphenyl)-2-quinazolinyl]amino}-3-methylphenyl)acrylamide
N120	N-(2-Methoxyphenyl)-2-[(4-nitrophenyl)sulfanyl]benzamide
N124	N-(2-aminophenyl)-4-[[[(8R,9S)-6-[(2R)-1-hydroxypropan-2-yl]-8-methyl-5-oxo-10-oxa-1,6,13,14-tetrazabicyclo[10.2.1]pentadeca-12(15),13-dien-9-yl]methyl-methylamino]methyl]benzamide
N130	4-[(2E)-1-Hydroxy-3-(4-hydroxyphenyl)-2-propen-1-yl]-1,2,3,5-benzenetetrol
N134	circumtrindene
N137	N-(2-Methoxyphenyl)-2-[(4-nitrophenyl)sulfanyl]benzamide

N141	2-(Cyclohexylamino)-2-oxoethyl 2-[(2-thienylsulfonyl)amino]benzoate
N144	(Z)-6'-Hydroxyferulate
N167	3-{1-[(2-AMINOPYRIDIN-4-YL)METHYL]INDOL-4-YL}-1-(5-BROMO-2-METHOXYPHENYL)UREA
N168	N-(2-{[6-(2,6-Dichloro-3,5-dimethoxyphenyl)-2-quinazolinyl]amino}-3-methylphenyl)acrylamide
N052	S-malonyl-4′-phosphopantetheine
P079	Eicosapentaenoic acid methyl ester
P118	Icosabutate
P299	1-(1Z-hexadecenyl)-sn-glycero-3-phosphocholine
N002	Murideoxycholic acid
N018	Glycoursodeoxycholic acid
N038	3-{[(2,3-Dihydroxypropoxy)(hydroxy)phosphoryl]oxy}-2-hydroxypropyl stearate
N042	(3β,5α)-Cholesta-8,24-dien-3-yl (9Z)-9-hexadecenoate
P199	g-Butyrobetaine
P234	N-Hydroxy-5-(methylsulfanyl)-L-norvaline
N033	DL-Arginine
N169	N(4)-phosphoagmatine
P119	Chitosan oligosaccharide lactate
P269	Pyrogallol-2-O-glucuronide
N097	2-O-Sulfo-α-L-idopyranuronic acid
P094	Prolylleucine
P007	4-Hydroxyhippuric Acid
P183	metachromin S
P348	3-Methylsulfolene
N127	Accelerator DM

Supplementary Table S2 List of microbe-metabolite correlations (Control)

Group	Genus	Metabolites
Control	*Campylobacter*	**Lipid:**<br>♦ Tetradecylamine<br>♦ Lauryldimethylamine oxide<br>**Carbohydrate:**<br>♦ 2-(3,4-Dihydroxyphenyl)-5,7-dihydroxy-4-oxo-4H-chromen-3-yl 4-O-β-D-glucopyranosyl-β-D-glucopyranosiduronic acid<br>♦
	Lachnoclostridium	**Amino Acid:**<br>♦ 4-Hydroxy-5-(2-hydroxy-2-propanyl)-2-methylbicyclo[3.1.0]hex-2-yl hexopyranoside<br>**Carbohydrate:**<br>♦ 2-(3,4-Dihydroxyphenyl)-5,7-dihydroxy-4-oxo-4H-chromen-3-yl 4-O-β-D-glucopyranosyl-β-D-glucopyranosiduronic acid<br>♦ [3-(3,4-methylenedioxyphenyl)-2-(mercaptomethyl)-1-oxoprolyl]glycine
	Moryella	**Lipid:**<br>♦ Lauryldimethylamine oxide<br>**Carbohydrate:**<br>♦ 2-(3,4-Dihydroxyphenyl)-5,7-dihydroxy-4-oxo-4H-chromen-3-yl 4-O-β-D-glucopyranosyl-β-D-glucopyranosiduronic acid

Supplementary Table S3 List of micorbe-metabolite correlations (LeanAd)

Group	Genus	Metabolites
LeanAd	*Allisonella*	**Lipid:**<br>♦ Icosabutate<br>♦ 1-(1Z-hexadecenyl)-sn-glycero-3-phosphocholine<br>♦ Murideoxycholic acid<br>**Carbohydrate:**<br>♦ Chitosan oligosaccharide lactate<br>♦ Pyrogallol-2-O-glucuronide<br>♦ 2-O-Sulfo-α-L-idopyranuronic acid
	Erysipelotrichaceae_UCG-006	**Lipid:**<br>♦ Icosabutate<br>♦ Murideoxycholic acid<br>**Amino Acid:**<br>♦ N-Hydroxy-5-(methylsulfanyl)-L-norvaline<br>**Carbohydrate:**<br>♦ Pyrogallol-2-O-glucuronide<br>♦ 2-O-Sulfo-α-L-idopyranuronic acid
	Lachnospiraceae_ND3007_group	**Lipid:**<br>♦ Icosabutate<br>♦ 1-(1Z-hexadecenyl)-sn-glycero-3-phosphocholine<br>♦ Murideoxycholic acid<br>**Amino Acid:**<br>♦ N-Hydroxy-5-(methylsulfanyl)-L-norvaline<br>**Carbohydrate:**<br>♦ g-Butyrobetaine<br>♦ Pyrogallol-2-O-glucuronide<br>♦ 2-O-Sulfo-α-L-idopyranuronic acid
